# Transcriptome profiling reveals divergent expression shifts in brown and white adipose tissue from long-lived GHRKO mice

**DOI:** 10.18632/oncotarget.5760

**Published:** 2015-09-21

**Authors:** Michael B. Stout, William R. Swindell, Xu Zhi, Kyle Rohde, Edward O. List, Darlene E. Berryman, John J. Kopchick, Adam Gesing, Yimin Fang, Michal M. Masternak

**Affiliations:** ^1^ Robert and Arlene Kogod Center on Aging, Mayo Clinic, Rochester, MN, USA; ^2^ Department of Dermatology, University of Michigan, Ann Arbor, MI, USA; ^3^ Center for Reproductive Medicine, Department of Obstetrics and Gynecology, Peking University Third Hospital, Beijing, China; ^4^ College of Medicine, Burnett School of Biomedical Sciences, University of Central Florida, Orlando, FL, USA; ^5^ Edison Biotechnology Institute and Heritage College of Osteopathic Medicine, Ohio University, Athens, OH, USA; ^6^ Department of Oncological Endocrinology, Medical University of Lodz, Lodz, Poland; ^7^ Geriatrics Research Laboratory, Department of Internal Medicine, Southern Illinois University School of Medicine, Springfield, IL, USA; ^8^ Department of Head and Neck Surgery, The Greater Poland Cancer Centre, Poznan, Poland

**Keywords:** brown adipose tissue, growth hormone, inflammation, metabolism, white adipose tissue, Gerotarget section

## Abstract

Mice lacking the growth hormone receptor (GHRKO) exhibit improved lifespan and healthspan due to loss of growth hormone signaling. Both the distribution and activity of brown and white adipose tissue (BAT and WAT) are altered in GHRKO mice, but the contribution of each tissue to age-related phenotypes has remained unclear. We therefore used whole-genome microarrays to evaluate transcriptional differences in BAT and WAT depots between GHRKO and normal littermates at six months of age. Our findings reveal a unique BAT transcriptome as well as distinctive responses of BAT to *Ghr* ablation. BAT from GHRKO mice exhibited elevated expression of genes associated with mitochondria and metabolism, along with reduced expression of genes expressed by monocyte-derived cells (dendritic cells [DC] and macrophages). Largely the opposite was observed in WAT, with increased expression of DC-expressed genes and reduced expression of genes associated with metabolism, cellular respiration and the mitochondrial inner envelope. These findings demonstrate divergent response patterns of BAT and WAT to loss of GH signaling in GHRKO mice. These patterns suggest both BAT and WAT contribute in different ways to phenotypes in GHRKO mice, with *Ghr* ablation blunting inflammation in BAT as well as cellular metabolism and mitochondrial biogenesis in WAT.

## INTRODUCTION

Growth hormone (GH) is a pivotal modulator of postnatal growth, metabolism, and adiposity in mammals. More recent studies have also determined that the magnitude of GH action in mammalian species is a major determinant of healthspan and in some cases longevity [1, 2]. Transgenic GH-overexpressing mice develop phenotypes associated with premature aging and have a shortened lifespan [3], while human acromegaly patients display greater disease burden and compressed life expectancy [4]. In contrast, Ames dwarf, Snell dwarf, and GH receptor-knockout (GHRKO) mice are protected from a variety of age-related diseases and live between 30-60% longer than their respective normal littermates [5-7]. The effects of genetic GH-deficiency and GH-resistance on human lifespan remains unclear, yet available evidence indicates these mutations confer protection from several age-related diseases including atherosclerosis, diabetes, and cancer [8–10]. Multiple interrelated mechanisms involving metabolic adjustments, reduced inflammation, and enhanced stress resistance contribute to the healthspan- and lifespan-extending effects of curtailed GH action [1, 2]. Interestingly, many of these mechanisms are closely linked with adipose tissue homeostasis, which is often perturbed with advancing age [11, 12].

Both white (WAT) and brown (BAT) adipose tissues undergo significant age-related functional changes in mice and humans [11, 12]. WAT, particularly subcutaneous depots, begin to display declines in preadipocyte replication and adipogenic potential in mid-to-late life [11, 13]. The aging process also promotes declines in BAT mass and activity [14–17], which may be a result of progenitor exhaustion, mitochondrial dysfunction, and/or perturbed endocrine control of brown adipocyte formation and function [18–20]. These declines are believed to be significant contributors to the age-related exacerbation of visceral adiposity, ectopic lipid deposition, and accompanying metabolic disorders [11, 12]. Long-lived GH-related mutant mice are protected from lipid redistribution and metabolic disorders with advancing age. We and others previously reported that Ames dwarf, Snell dwarf, and GHRKO mice preferentially deposit lipid in subcutaneous WAT depots, which is likely in response to the preservation of preadipocyte differentiation capacity [21–25]. Surprisingly, visceral WAT in GHRKO mice has a protective role by enhancing systemic insulin sensitivity and metabolic homeostasis [26], which lies in sharp contrast to other models [27, 28]. In alignment with these observations, BAT mass [29, 30] and thermogenesis-related gene expression also appear to be enhanced in GHRKO mice [29].

Although it is generally accepted that improvements in adipose tissue homeostasis in Ames dwarf, Snell dwarf, and GHRKO mice are mechanistically linked to reductions in inflammation and increased adiponectin expression [21, 22, 26, 31–33], it remains unclear if depot-specific changes occur and what role BAT modifications may play in this process. To address these questions, we evaluated transcriptional differences in WAT and BAT depots between GHRKO and normal littermates at six months of age. Our findings show that BAT and WAT have distinctive global gene expression profiles and that both types of adipose tissue respond differently to *Ghr* ablation. These response patterns involve modulation of inflammatory and metabolic pathways, with expression shifts in GHRKO BAT suggestive of decreased inflammation and heightened cellular metabolism. Our findings thus identify novel mechanisms by which BAT may mediate favorable effects of *Ghr* ablation on aging, and illustrate how such mechanisms may contrast sharply with those involving WAT.

## RESULTS

### BAT expression profiles are distinct from WAT and show divergent responses to *Ghr* ablation

**Figure 1:**
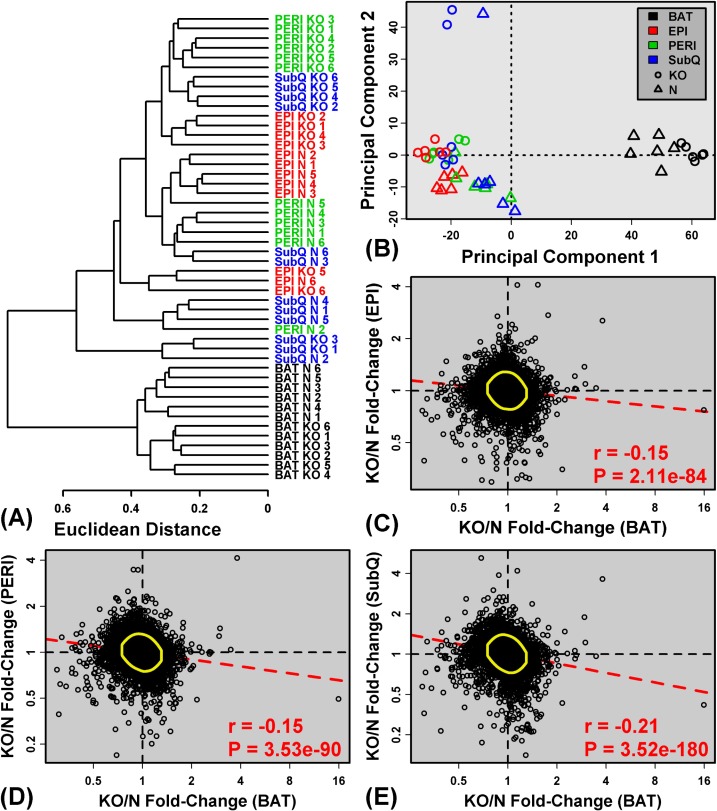
BAT shows distinct expression patterns and divergent expression shifts in GHRKO as compared to normal mice **A.** The 48 samples were clustered based upon expression of 18,124 genes with detectable expression in at least 33% of samples (16 of 48 samples). The Euclidean distance between expression profiles was calculated and hierarchical clustering was performed using average linkage. **B.** Principal components plot. Samples were plotted with respect to the first two principal components extracted from the normalized expression matrix (18,124 genes × 48 samples). Parts **C. - E.** compare estimated fold-changes (KO/N) in BAT with those from **C.** EPI, **D.** PERI and **E.** SubQ. Each point represents an individual gene expressed in both depots being compared. Yellow circles encompass the 90% of genes closest to the bivariate mean (Mahalanobis distance), and the dashed red line represents the robust linear model fit. The Spearman correlation coefficient and associated *p*-value are listed in the lower right corner of **C. - E.**.

We used *in situ* oligonucleotide whole-transcript arrays to profile gene expression in 48 adipose tissue samples from GHRKO (KO) and normal (N) littermate mice. An initial cluster analysis revealed no outliers among the 48 samples, but it was immediately clear that BAT samples were distinct from WAT depots (SubQ, EPI and PERI), regardless of genotype (Figure 1A). This same pattern was discernable when all samples (BAT + WAT) were plotted with respect to the first two principal component axes (Figure 1B). BAT expression profiles were thus distinct from all WAT depots analyzed. Among WAT depots, differences in gene expression profiles were less pronounced (Figures 1A and 1B), and in general, genotype (KO or N) had a stronger impact on WAT than anatomical location. However, cluster and principle components analyses of WAT samples alone (excluding BAT) revealed some distinction between EPI and SubQ, with PERI samples showing an intermediate “EPI/SubQ” expression profile (Supplemental Figure 1). The EPI/SubQ distinction was stronger in normal mice and attenuated in GHRKO animals (Supplemental Figure 1). For all WAT depots, however, fold-changes (KO/N) followed a moderate, yet significant, negative genome-wide correlation with those calculated for BAT (Figure 1C-1E). Global effects of *Ghr* ablation on gene expression in BAT thus tended to be opposite of those in WAT.

**Figure 2:**
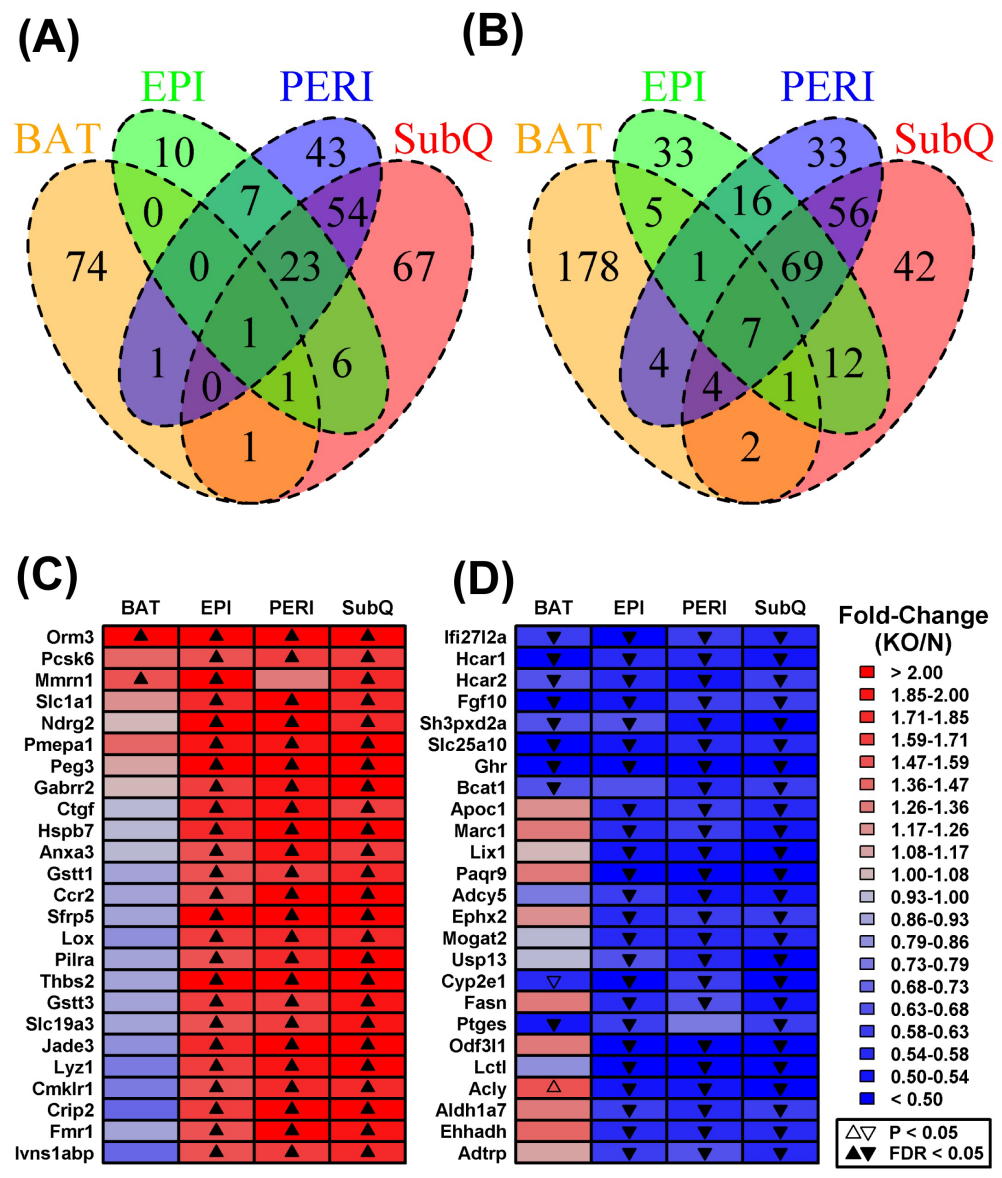
Shared or partially shared BAT/WAT expression changes in GHRKO mice as compared to normal controls We identified 346, 319, 280 and 192 DEGs with respect to subcutaneous (SubQ), renal (PERI), brown (BAT) and epididymal fat (EPI) (FC > 1.50 or FC < 0.67 with FDR < 0.05). Venn diagrams show the numbers of shared and depot-specific DEGs either **A.** increased or **B.** decreased in GHRKO mice as compared to normal controls. Heatmaps show genes most consistently **C.** increased or **D.** decreased across all four depots. Genes were primarily sorted according to the number of depots in which they were differentially expressed (FC > 1.50 or FC < 0.67 with FDR < 0.05) and then secondarily sorted according to estimated fold-change (KO/N). The 25 top-ranked genes most consistently altered across fat depots are shown in **C.** and **D.**.

Differential expression analysis was next performed to identify genes with significantly altered expression in GHRKO mice with respect to each adipose depot (Supplemental Table 1). The largest number of DEGs was found in SubQ WAT (153 increased; 193 decreased), followed by PERI WAT (129 increased; 190 decreased), BAT (78 increased; 202 decreased), and EPI WAT (48 increased; 144 decreased) (Figures 2A and 2B). Consistent with the above trends (Figure 1), many DEGs were shared among WAT depots, but few of these also overlapped with those identified in BAT (Figures 2A and 2B). For instance, aside from *Ghr*, only six genes were significantly decreased in all adipose tissue depots analyzed (*Ifi27l2a*, *Hcar1*, *Hcar2*, *Fgf10*, *Sh3pxd2a*, *Slc25a10;* Figure 2D). Only a few genes, moreover, were significantly elevated in BAT as well as one or more WAT depots (e.g., *Orm3*, *Mmrn1*, *Tc2n*, *Gm10419*; Figure 2C). More genes, however, could be identified with BAT-specific or WAT-specific changes in gene expression (Figures 2A and 2B). For instance, 74 DEGs were increased only in BAT (Figure 2A), while 178 DEGs were decreased only in BAT (Figure 2B). Analysis of DEGs again indicated that *Ghr* ablation elicits unique gene expression shifts in BAT as compared to WAT, consistent with trends observed at the global level of genome-wide expression (Figure 1A).

### *Ghr* ablation modulates BAT metabolic and inflammatory gene expression profiles

**Figure 3:**
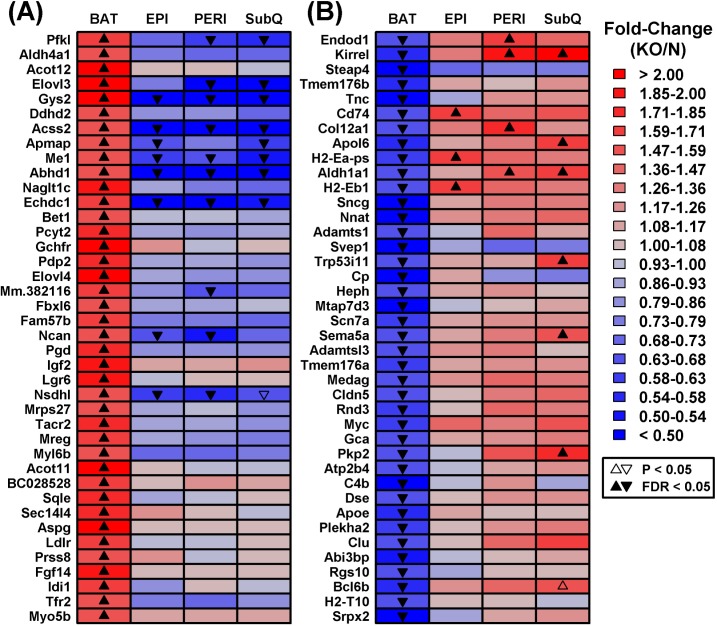
Genes with divergent gene expression changes in BAT *versus* WAT (GHRKO versus normal mice) The 17,831 genes expressed in BAT and each WAT depot were filtered to identify those showing the most divergent gene expression changes in BAT as compared to WAT. **A.** DEGs increased in BAT and decreased/unaltered in WAT. GHRKO-increased genes (BAT) were filtered based upon the difference in fold-change estimated for BAT and the largest fold-change estimated for any WAT depot (log_2_ scale). **B.** DEGs decreased in BAT and increased/unaltered in WAT. GHRKO-decreased genes (BAT) were ranked based upon the difference in fold-change estimated for BAT and the largest fold-change estimated for any WAT (log_2_ scale). The 40 top-ranked genes with divergent expression shifts in BAT and WAT are shown in **A.** and **B.**

We identified a number of genes divergently altered by *Ghr* ablation in BAT and WAT depots. These included genes increased in BAT from GHRKO mice, despite being uniformly decreased in WAT depots (e.g., *Gys2*, *Acss2*, *Me1*, *Abhd1, Echdc1*; Figure 3A). Gene ontology (GO) biological process (BP) terms enriched among such genes were disproportionately related to metabolism, including organic acid metabolic process, oxidation-reduction process, cholesterol biosynthesis, and positive regulation of lipid & acetyl-CoA metabolic processes (Supplemental Figure 2A). Conversely, we also identified genes with reduced expression in BAT from GHRKO mice and increased expression in at least one WAT depot (e.g., *Endod1*, *Kirrel*, *Cd74*, *Col12a1, Apol6, H2-Eb1, Trp53i11, Sema5a, Pkp2*; Figure 3B). Such genes were disproportionately associated with immune and inflammation-related GO BP terms such as regulation of immune system process, cell motility and activation, innate immune response, inflammatory response, and wound healing (Supplemental Figure 2B).

### *Ghr* ablation curtails WAT expression of mitochondria-associated genes

**Figure 4:**
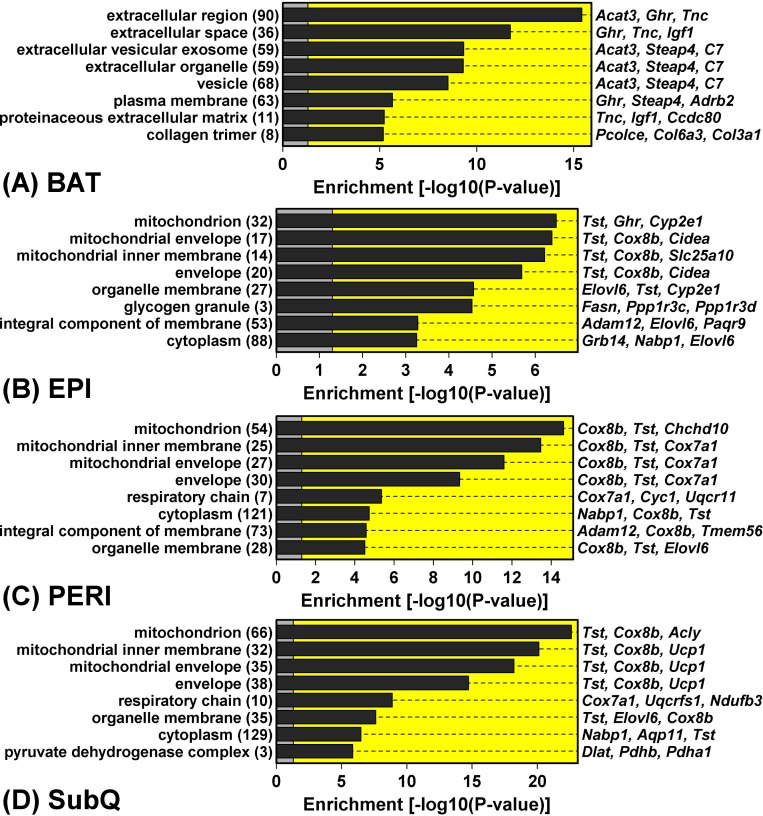
Genes with GHRKO-decreased expression in WAT (but not BAT) are associated with mitochondria and the mitochondrion inner envelope GHRKO-decreased DEGs were identified with respect to each fat depot and analyzed to identify significantly overrepresented GO cellular component (CC) terms. The top-ranked eight most enriched GO CC terms are listed for each depot. The number of GHRKO-decreased DEGs associated with each GO CC term is listed (left margin, within parentheses) along with exemplar GHRKO-decreased DEGs (right margin).

**Figure 5:**
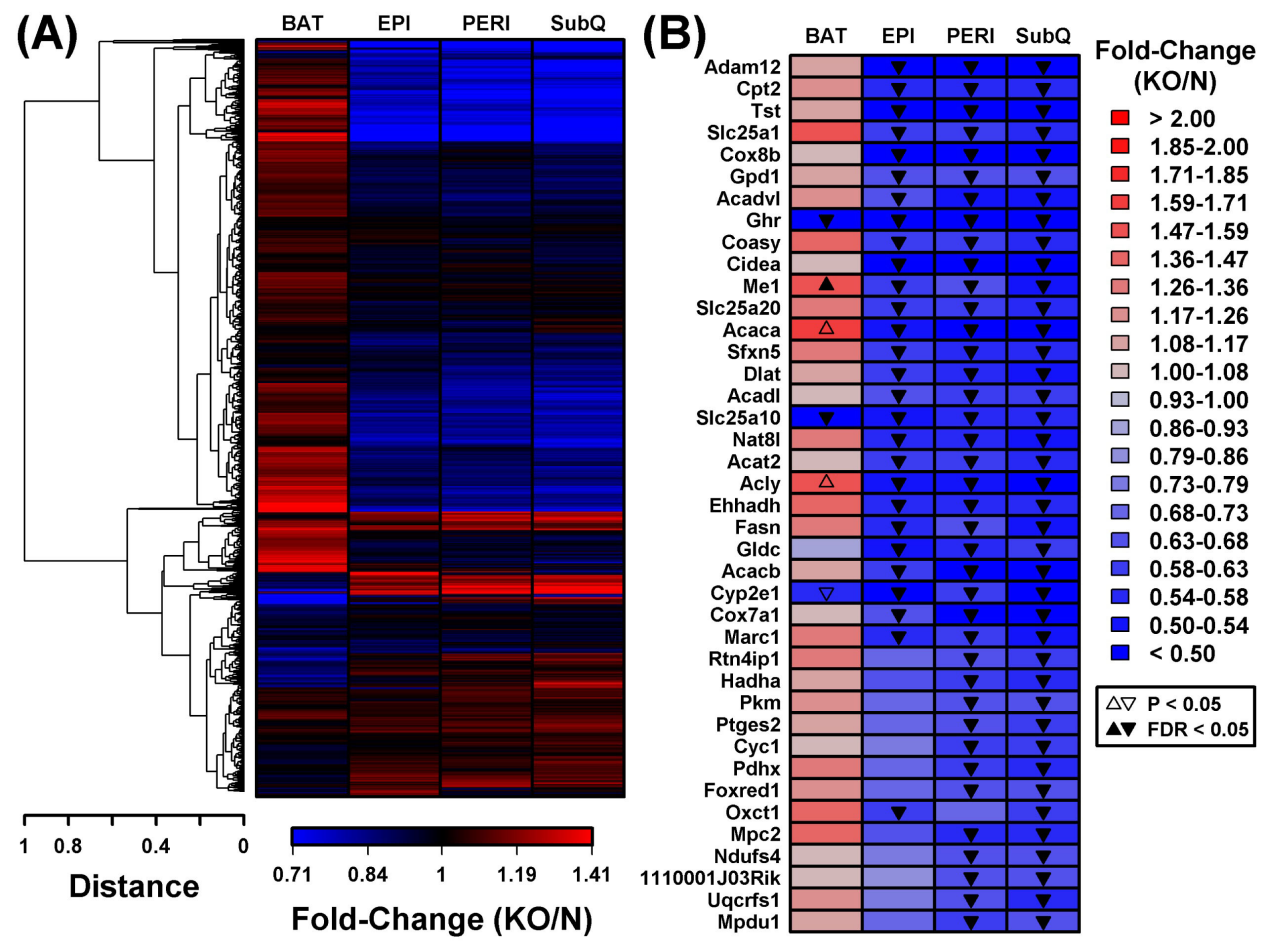
BAT and WAT show divergent mitochondrial gene expression changes in GHRKO as compared to normal controls **A.** Cluster analysis of 1,450 BAT/WAT-expressed genes associated with the “mitochondrion” GO BP term (GO:0005739). Complete linkage hierarchical clustering was performed based upon the Euclidean distance between fold-change estimates (BAT, EPI, PERI and SubQ). **B.** Mitochondrion genes (GO:0005739) most consistently decreased in WAT from GHRKO mice. The 1,450 genes were primarily sorted to identify those most consistently differentially expressed in WAT depots (FC < 0.67 with FDR < 0.05) and secondarily sorted based upon fold-change (i.e., largest fold-change estimated for any WAT depot). The 40 top-ranked genes based upon these criteria are shown.

In contrast to the robust increase in metabolism-related gene expression in BAT from GHRKO mice, WAT depots all displayed reduced expression of genes associated with mitochondria (Figure 4). DEGs with decreased expression in WAT, for instance, were significantly enriched with genes associated with mitochondrion, mitochondrial envelope, and mitochondrial inner membrane (Figure 4B-4D). This was not the case, however, for DEGs with decreased expression in BAT, which were instead associated with extracellular proteins (Figure 4A). Cluster analysis of 1,450 genes associated with the mitochondrion GO CC term (GO:0005739) revealed a distinct group of genes with decreased expression in all WAT depots, even though their expression was unaltered or elevated in BAT (Figure 5A). Examples of such mitochondrion genes included *Cpt2*, *Tst*, *Slc25a1* and *Cox8b*, each of which was decreased in all WAT depots without corresponding changes in BAT (Figure 5B). Many other mitochondrion genes were decreased in at least two WAT depots and not similarly altered in BAT (Figure 5B).

### Anti-inflammatory gene expression changes in BAT from GHRKO mice: evidence for reduced dendritic cell and macrophage infiltration

**Figure 6:**
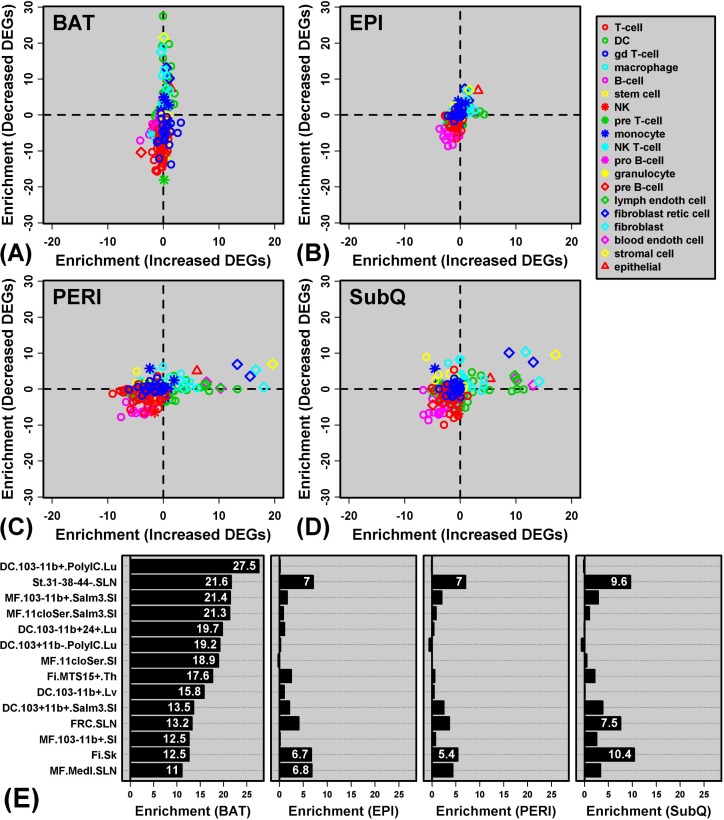
Genes with decreased expression in BAT are enriched with genes specifically expressed by dendritic cells and macrophages (Immunological Genome Project) **A. - D.**GRHKO-increased and -decreased DEGs were screened with respect to 222 cell populations included within the Immunological Genome Project expression database. Enrichment statistics were calculated for each cell population and with respect to GHRKO-increased DEGs (horizontal axis) and GHRKO-decreased DEGs (vertical axis). For each cell population, enrichment statistics represent −log_10_-transformed *p*-values derived from the test of whether DEGs are disproportionately included among “signature genes” with a cell type-specific expression pattern (Wilcoxon rank sum test). Positive statistics indicate that DEGs are enriched with genes specifically expressed by a given cell type, while negative statistics indicate that DEGs are enriched with genes showing a non-specific expression pattern. In **A. - D.**, each point represents an individual cell type, with different symbols for the main cell type categories included within the Immunological Genome Project (see legend). **E.** Cell populations for which signature genes most strongly overlap with GHRKO-decreased DEGs identified in BAT (DC = dendritic cell; st = stromal cell; MF = macrophage; Fi = fibroblast). Further information on each cell type is available online (http://www.immgen.org/). For each cell population, enrichment statistics (−log_10_-transformed p-values) are shown with respect to BAT, EPI, PERI and SubQ fat, respectively.

Genes specifically decreased in BAT from GHRKO mice were associated with immune and inflammatory GO BP terms (Supplemental Figure 2B). One possible explanation for this pattern is that *Ghr* ablation leads to reduced immune cell infiltration of BAT in GHRKO mice, resulting in decreased expression of genes characteristically expressed by particular immune cell subsets. To address this possibility, we utilized the Immunological Genome Project (IGP) database [38] to determine whether genes with decreased expression in BAT included “signature genes” expressed primarily by cell types from the innate or adaptive immune systems (Figure 6).

GHRKO-decreased DEGs in BAT were significantly enriched with genes specifically expressed by DCs and macrophages (Figures 6A and 6E). For instance, GHRKO-decreased DEGs in BAT included *H2-Ea-ps*, *C3ar1*, *Cxcl9*, *C7*, *C1qb* and *Ifi205*; each of these genes was expressed at significantly higher levels in lung-derived DCs relative to all other cell types in the IGP database (i.e., IGP population DC.103-11b+.PolyIC.Lu; Figure 6E and Supplemental Figure 3A). Overall, GHRKO-decreased DEGs in BAT were significantly more likely to be specifically expressed by this DC population, as compared to all other BAT-expressed genes (*P* = 3.01 × 10^−28^; Wilcoxon rank sum test; Supplemental Figure 3B).

We repeated the same analysis in WAT but observed much weaker enrichment of immune cell-specific genes among GHRKO-decreased DEGs (Figures 6B-6E). GHRKO-decreased DEGs, for instance, showed no enrichment for genes specifically expressed by lung-derived DCs (P ≥ 0.727; Supplemental Figure 3C-3E). To the contrary, GHRKO-increased DEGs were modestly enriched for genes specifically expressed by DCs (PERI and SubQ; Figures 6C and 6D), suggesting possibly increased inflammatory status of these WAT depots. Interestingly, DEGs with elevated expression in WAT (PERI and SubQ) tended to be expressed by non-immune cell types, including stromal cells, fibroblastic reticular cells, skin fibroblasts, and blood endothelial cells (Figures 6C and 6D). For example, GHRKO-increased DEGs in SubQ were expressed at significantly elevated levels in blood endothelial cells as compared to all other IGP cell types (e.g., *Akr1c14*, *Palmd*, *Shroom4*, *Prex2*, *Sorbs2*; *P* = 8.08 × 10^−14^). These observations suggest increased DC infiltration of WAT in GHRKO mice, accompanied by heightened vascularity and possibly altered adipocyte differentiation status (PERI and SubQ).

## DISCUSSION

Curtailed GH action elicits beneficial effects on age-related diseases through a variety of mechanisms [1, 2]. Many of the enhancements observed in long-lived GH-related mutants are often associated with the preservation of adipose tissue homeostasis with advancing age [11, 12]. Our previous reports have established that subcutaneous WAT lipid storage capacity remains intact in GHRKO mice into late-life, which likely prevents age-related lipid redistribution and metabolic dysfunction [22–25]. Visceral WAT in GHRKO mice has also been reported to enhance metabolic homeostasis through alterations in lipid metabolism and adipokine production [26], which is certainly contrary to traditional dogma [27, 28]. Although fewer studies have evaluated the effects of *Ghr* ablation on BAT activity, GHRKO mice have been reported to have greater BAT mass [29, 30] and increased expression of genes responsible for thermogenic activity [29]. These studies suggest GH may differentially modulate adipose tissue phenotypes in a depot-specific manner. Given that transcriptional alterations are likely associated with the previously described adipose tissue functional changes in GHRKO mice, we evaluated transcriptome profiles in WAT and BAT to determine if the beneficial effects associated with *Ghr* ablation is more closely aligned with specific depots.

Our analysis revealed distinct BAT and WAT expression profiles and divergent responses of each adipose type to *Ghr* ablation. Consistent with this, previous reports have also shown that WAT and BAT possess intrinsically different transcriptomes [39, 40], likely due to differences in progenitor lineage [41], resident immune cells [42], and metabolic phenotypes [40]. Our findings extend this work and demonstrate that BAT and WAT respond differently to the altered *in vivo* endocrine status of GHRKO mice. This differential response involved genes associated with cellular respiration and metabolism, with GHRKO BAT showing increased expression of mitochondrial genes and GHRKO WAT featuring decreased expression of such genes. These expression shifts mirror quintessential differences in BAT *versus* WAT physiology. Whitening of adipose tissue during the early postnatal period in sheep, for instance, is characterized by decreased mitochondrial abundance and expression of genes involved in mitochondrial function [43]. This may underlie physiological adipose shifts in GHRKO mice, evidenced in previous reports by enhanced BAT metabolism [29] and reduced WAT metabolism [22–24]. Such shifts may be connected to GHRKO aging and longevity in two ways. First, heightened BAT metabolism could improve energy dissipation, in some ways mimicking systemic consequences of low-calorie diets [44, 45], while leading to improvements in glucose tolerance and insulin sensitivity [46]. Secondly, dampening metabolic processes in WAT may favorably impact oxidative stress levels [47], but perhaps more importantly, could reduce WAT-derived pro-inflammatory cytokines (e.g., IL-6 and TNF-α) [48].

GHRKO mice appear resistant to some favorable effects of caloric restriction (CR). For instance, GHRKO mice do not exhibit increased lifespan when provided a CR diet [49, 50]. Additionally, key expression markers of mitochondrial biogenesis are not further enhanced by CR in GHRKO mice [51]. These important observations suggest CR and GH signaling repression improve longevity through mechanisms that at least partially overlap. In this context, it is interesting to note that an earlier proteomic study of rats has also identified divergent responses of BAT and WAT to CR [52]. In contrast to our findings, however, CR increased biomarkers of mitochondrial activity in WAT, but did not alter or decreased the same biomarkers in BAT [52]. These findings are contrasting with our own observations. This discrepancy may be due to our focus on the transcriptome, rather than the proteome, or by the fact that we studied mice, rather than rats. Alternatively, our findings and those of earlier work [52] may reflect a key difference between two well-studied aging interventions (CR and loss of GH signaling), wherein these two interventions have divergent, but opposite, effects on BAT and WAT metabolism. In some respects, this is not surprising, since CR and loss of GH action have opposing effects on systemic adiposity, with CR tending to reduce total adipose mass and curtailed GH signaling tending to increase it [22, 23, 49]. In future work, it may be valuable to compare CR responses of BAT and WAT in GHRKO and normal mice. For instance, assuming that CR and *Ghr* ablation have additive effects, we predict that BAT metabolism would be highest in *ad lib*-fed GHRKO mice, lowest in CR-fed normal mice, and intermediate in both *ad lib*-fed normal and CR-fed GHRKO mice.

Immune cells reside in adipose tissue and interactions involving these cell types play a significant role in modulating systemic metabolism and inflammatory status [42, 53, 54]. Genes with decreased expression in BAT were disproportionately associated with immunity and inflammation response (Supplemental Figure 2), suggesting attenuated BAT immune cell infiltration in GHRKO mice. Given the close associations between inflammation and mitochondrial dysfunction [55], we used data from the Immunological Genome Project [38] to computationally discern the identity of immune cell types contributing to this pattern [56–58]. This showed that genes with decreased expression in GHRKO BAT were often expressed by DC and macrophage populations. Both of these monocyte-derived cell types are commonly associated with adipose tissue and tend to become more numerous in adipose as a result of obesity [59]. This inflammatory process has been best characterized in WAT (rather than BAT), although prior studies have identified increased BAT macrophage numbers in obese mouse genotypes [60]. DCs are also commonly associated with adipose tissue, particularly in the obese state, and their presence appears to facilitate development of Th17 responses and obesity-associated insulin resistance [61, 62]. In GHRKO mice, reduced BAT infiltration by macrophages and/or DCs may dampen systemic inflammation with aging and contribute to insulin sensitivity. These immune cell types, moreover, are reported to have deleterious effects on adipose metabolism [54]. Their reduced prominence in GHRKO BAT could contribute to the apparent enhancement of BAT mitochondrial activity discussed above. Interestingly, the same analysis in WAT revealed that immune cell populations were affected to a far lesser extent by *Ghr* ablation (Figure 6). In fact, in WAT we detected increased expression of genes expressed by non-immune cell types, particularly cells of stromal and fibroblastic origin. This may reflect a pro-proliferative and stem cell-like phenotype of GHRKO WAT, which may account for the greater progenitor differentiation capacity previously observed in young [63] and old adult GHRKO mice [24, 25]

Our analyses identified strong differences between BAT and WAT expression profiles, although we could also discern more subtle differences among the three WAT depots. At a genome-wide level, for instance, we could identify distinctive expression signatures for EPI and SubQ, even though each of these overlapped with and could not clearly be distinguished from PERI (Supplemental Figure 1). This result resonates with other studies that have documented transcriptional differences between visceral and subcutaneous WAT depots in rodents and humans [64–66]. It is interesting to note that EPI *versus* SubQ expression differences were relatively greater in controls and weaker in GHRKO mice (Supplemental Figure 1). The fact that the EPI/SubQ WAT distinction was weakened in GHRKO mice may reflect a unique aspect of WAT physiology in response to *Ghr* ablation. Previously, we showed that removal of visceral fat from normal mice improves insulin sensitivity and glucose homeostasis; however, these same benefits were absent when the same depots were removed from GHRKO animals [26]. Therefore, in GHRKO mice, visceral WAT physiology may more closely resemble SubQ and not generate the same deleterious factors that shorten lifespan in normal mice. Physiological and functional studies will thus be needed in future work to better understand whether changes in adipose tissue are mechanistically linked to the improved healthspan and lifespan of GHRKO mice.

In summary, our study demonstrates that BAT and WAT depots respond to *Ghr* ablation in divergent manners. We found that BAT and WAT respond by modulating metabolic and inflammatory pathways in opposing fashions, with expression shifts in BAT suggestive of decreased inflammation and heightened cellular metabolism in GHRKO mice. Our findings point towards new mechanisms by which BAT may mediate favorable effects of *Ghr* ablation on aging, healthspan and lifespan, and identify several ways in which the physiological balance of BAT and WAT activity is perturbed with systemic loss of GH sensitivity.

## MATERIALS AND METHODS

### Animals and tissue collection

GHRKO and normal (N) littermate mice (GHR+/−; *N* = 6/group; heterogeneous background) were maintained at Southern Illinois University [34]. All mice were housed 4-5 animals/cage at 22 ± 0.5°C on a 12:12-hour light-dark cycle. Mice had *ad libitum* access to standard laboratory chow and water. At six-months of age, mice were anesthetized with isoflurane and euthanized by cervical dislocation prior to dissection. WAT depots including inguinal (SubQ), epididymal (EPI), and perirenal (PERI) along with BAT were immediately excised, flash-frozen, and stored at ^−^80°C for future analyses. All procedures were approved by the Institutional Animal Care and Use Committee of Southern Illinois University.

### RNA extraction

RNA was extracted from ~50 mg of frozen adipose tissues using RNeasy mini kits (Qiagen, Valencia, CA) according to the manufacturer’s protocol including on column DNase treatment to eliminate any DNA contamination. Samples were homogenized with zirconium oxide beads using a Bullet Blender Homogenizer (Next Advance, Averill Park, NY). Once homogenized adipose tissues were centrifuged at 12,000 x g for 10 min at 4°C and the upper lipid layer was excluded by transferring the supernatant to a new tube. Pure RNA was then extracted and eluted into 30 μl of nuclease-free water. RNA concentrations and purities were determined using an Epoch plate reader (BioTek, Winooski, VT). RNA samples with an A260/A280 ratio < 1.9 were re-extracted. Extracted RNA was stored at ^−^80°C in preparation for microarray analyses.

### Microarray analyses

RNA quality was assessed by the Agilent Bioanalyzer Nano chip (Agilent Technologies, Santa Clara, CA). Labeled single-stranded cDNA (sscDNA) was generated using 400ng of total RNA according to Whole-transcript (WT) Sense Target Labeling Assay protocol using Ambion’s WT Expression kit and GeneChip WT Terminal Labeling kit. Labeled sscDNA (5.5 μg) was then hybridized onto GeneChip Mouse Gene 1.0 ST Array (Affymetrix, Santa Clara, CA), which analyzes 28,853 gene transcripts using ~760,000 probe sets (on average 27 probes per gene). Staining and washing of the arrays were conducted using a Fluidics 450 station, and scanned using Affymetrix GeneChip Command Console Software (AGCC) and GeneChip® Scanner 3000 7G. All these procedures were conducted according to the manufacturer’s instructions.

### Bioinformatics and statistics

Normalized gene expression values were calculated for the 48 arrays using the robust multichip average (RMA) algorithm (R package: oligo; R function: rma) [35]. This generated log_2_-normalized expression values for 35,556 “transcript clusters”, with each cluster incorporating probes targeting one of 20,862 mouse genes (i.e., the “core” option was used with the oligo package rma function). For each sample, transcript clusters with expression scores beneath the 20th percentile were considered undetected. Based on this threshold, 28,445 clusters were detected (present) and 7,111 clusters were undetected (absent) in each sample. For subsequent analyses, a representative transcript cluster was chosen for each mouse gene, with the representative chosen as the cluster with highest average expression across all 48 arrays included in the analysis. This limited analyses to 20,862 clusters (one representative for each mouse gene). For differential expression testing, we further limited analyses to only those genes for which the associated transcript cluster was detected with respect to at least 3 of the 12 arrays analyzed for a given fat depot. This criterion yielded between 18,081 - 18,285 genes for each fat depot. These fat-expressed genes were subsequently analyzed for differential expression (GHR−/− vs. control) using linear models with empirical Bayes moderated t-statistics (R package: limma; R functions: lmFit and eBayes) [36]. To correct for multiple hypothesis testing among the 18,081 - 18,285 genes analyzed with respect to a given depot, raw *p*-values associated with moderated t-statistics were adjusted using the Benjamini-Hochberg method to control the false discovery rate (R function: p.adjust) [37]. Differentially expressed genes (DEGs) were identified with respect to each fat depot, respectively, based upon a fold-change (FC) threshold of 1.50 (or 1/1.50 = 0.67) and an FDR-adjusted *p*-value of 0.05.

## SUPPLEMENTARY MATERIAL FIGURES AND TABLES





## Abbreviations

AGCC: Affymetrix GeneChip Command Console
BAT: brown adipose tissue
BP: biological process
CC: cellular component
CR: calorie restriction
DC: dendritic cells
DEG: differentially expressed genes
EPI: epididymal
FC: fold-change
FDR: false discovery rate
Fi: fibroblast
GH: growth hormone
GHRKO: growth hormone receptor knockout
GO: gene ontology
IGP: Immunological Genome Project
KO: knockout
MF: macrophage
N: normal littermates
PERI: perirenal
RMA: robust multichip average
sscDNA: single-stranded cDNA
st: stromal cell
SubQ: inguinal
WAT: white adipose tissue
WT: whole-transcript
