# Supplementary material for: Transcriptome profiling reveals divergent expression shifts in brown and white adipose tissue from long-lived GHRKO mice
Source: Oncotarget. 2015 Sep 21;6(29):26702–15. doi: 10.18632/oncotarget.5760 (PMC4694946; doi:10.18632/oncotarget.5760)
Supplement: Supplementary file 1 [file oncotarget-06-26702-s001.pdf]

Transcriptome profiling reveals divergent expression shifts in brown and white adipose tissue from long-lived GHRKO mice

Supplementary Material

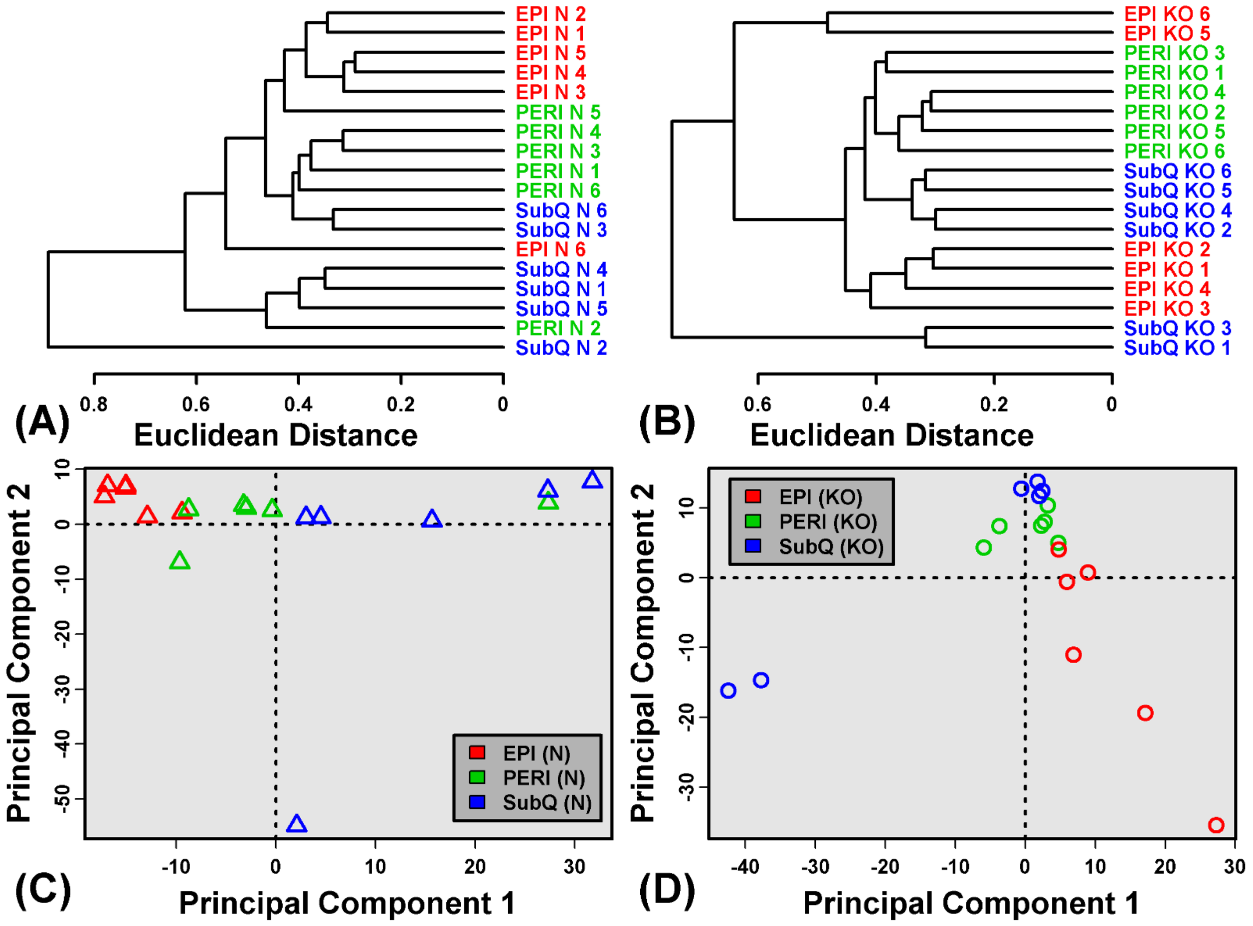

Supplemental Figure 1: Gene expression profiles of WAT depots are partially distinct and partially overlapping. A. The 18 WAT samples from normal mice were clustered based upon expression of 18,151 genes with detectable expression in at least 33% of samples (i.e., 6 of the 18 samples). The Euclidean distance between expression profiles was calculated and hierarchical clustering was performed using average linkage. **B.** The 18 WAT samples from GHRKO mice were clustered based upon expression of 18,016 genes with detectable expression in at least 33% of samples (i.e., 6 of the 18 samples). Euclidean distance between expression profiles was calculated and hierarchical clustering was performed using average linkage. **C.** Principal components plot. WAT samples from normal mice were plotted with respect to the first two principal components extracted from the normalized expression matrix (18,151 genes × 18 samples). **D.** Principal components plot. WAT samples from GHRKO mice were plotted with respect to the first two principal components extracted from the normalized expression matrix (18,016 genes × 18 samples).

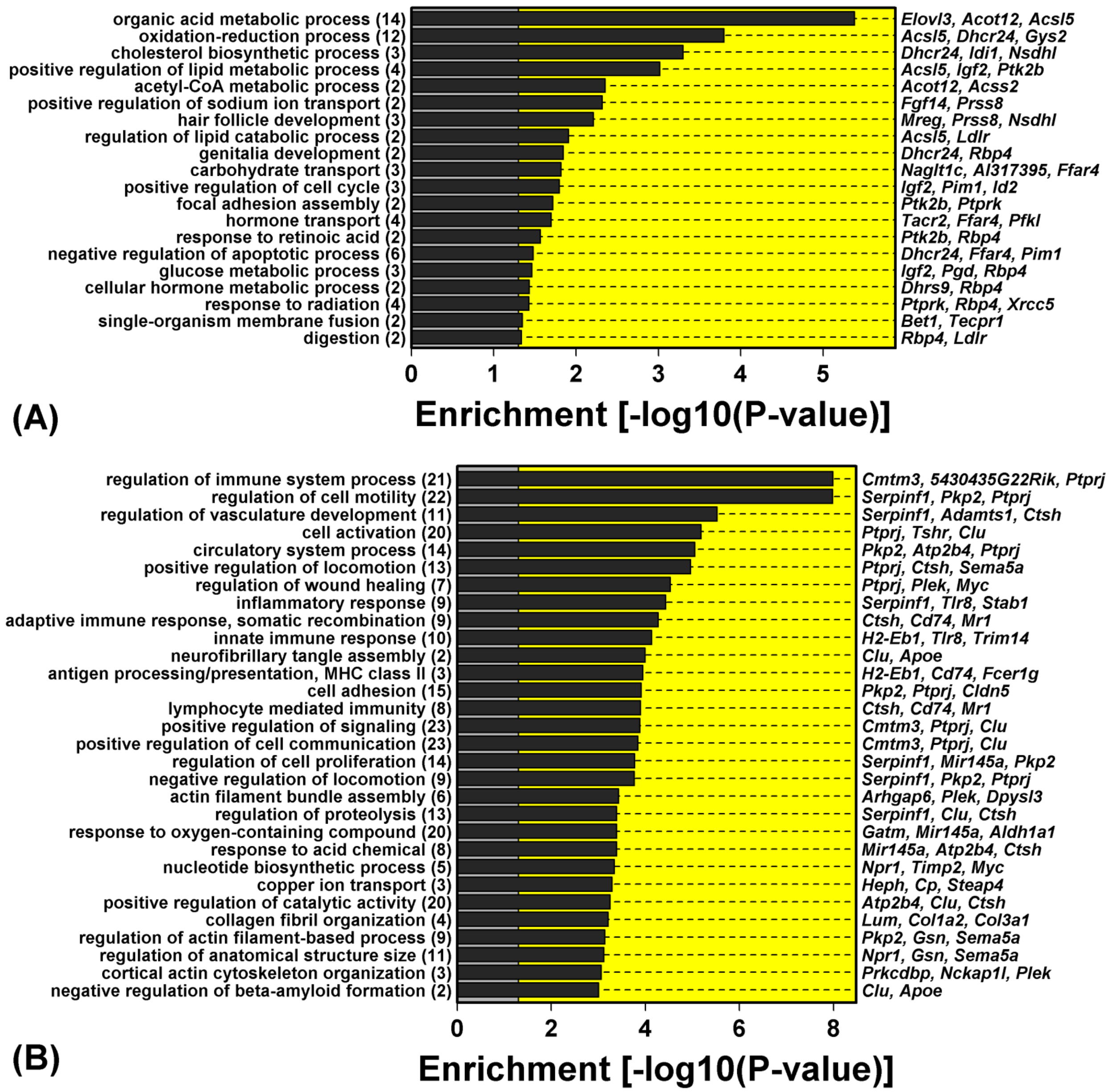

Supplemental Figure 2: Gene ontology (GO) biological process (BP) terms significantly overrepresented among genes altered by GHRKO specifically in BAT: A. We identified 73 GHRKO-increased genes in BAT (FC > 1.50 with FDR < 0.05) that were not significantly altered in WAT depots (P > 0.05 for each depot, respectively). These 73 genes were analyzed to identify overrepresented GO BP terms (compared to a background set of 17,758 other genes with detectable expression in all fat depots). The figure lists top-ranked GO BP terms with the strongest enrichment. **B.** We identified 174 GHRKO-decreased genes in BAT (FC < 0.67 with FDR < 0.05) that were not significantly altered in WAT depots (P > 0.05 for each depot, respectively). These 174 genes were analyzed to identify overrepresented GO BP terms (compared to a background set of 17,657 other genes with detectable expression in all fat depots). The figure lists top-ranked GO BP terms with the strongest enrichment. In both **A.** and **B.**, the number of BAT-specific DEGs associated with each GO term is indicated (left margin,

within parentheses). Exemplar BAT-specific DEGs associated with each GO term are also listed (right margin).

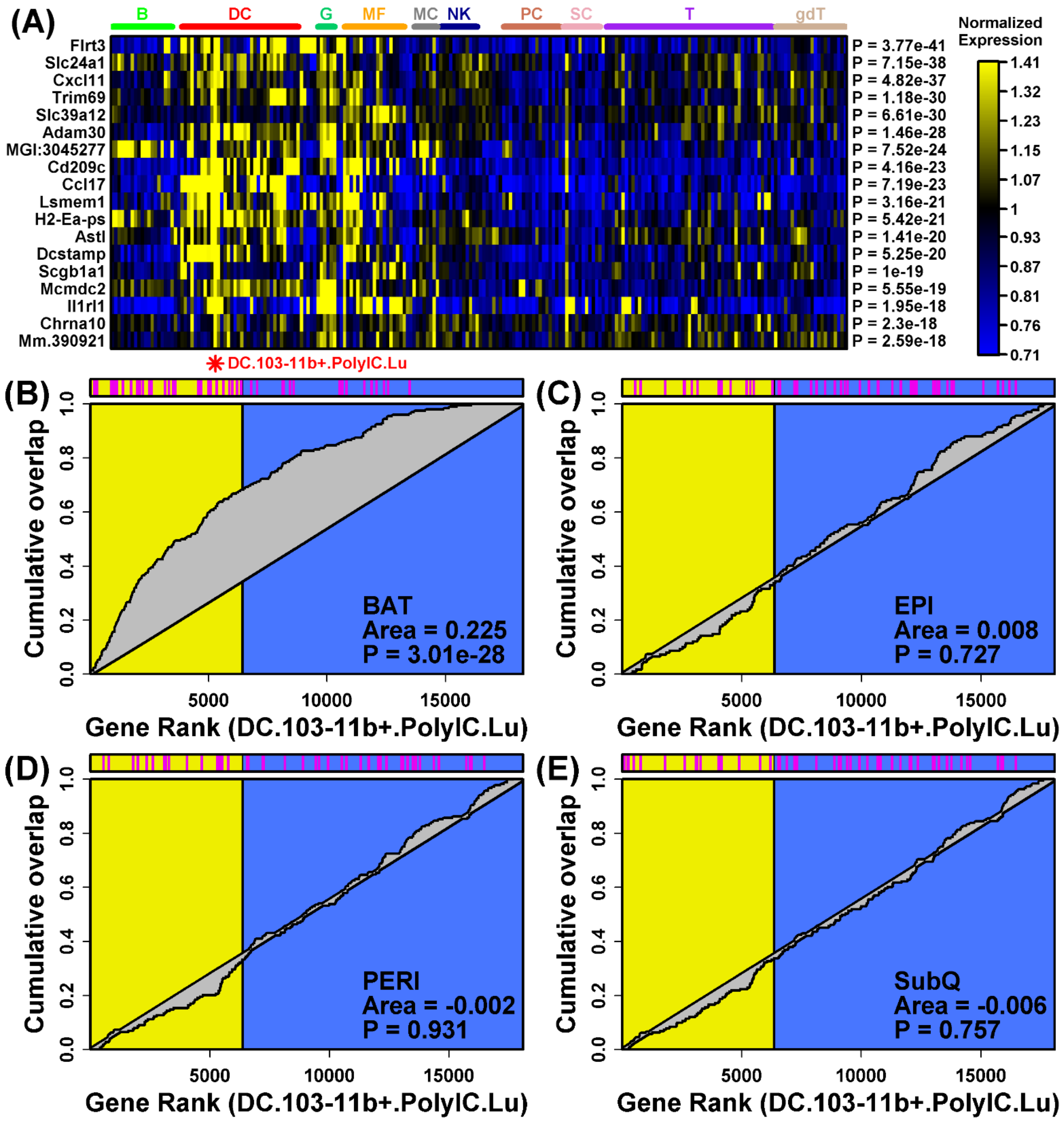

Supplemental Figure 3: GHRKO-decreased DEGs in BAT are enriched with genes specifically expressed by lung-derived dendritic cells (DC.103-11b+.PolyIC.Lu). A. The heatmap shows the 18 genes most specifically expressed in lung-derived dendritic cells (IGP identifier DC.103-11b+.PolyIC.Lu). Expression of each gene is shown across the 222 IGP cell populations, with expression normalized to the gene's average expression across all cell types (B = B-cells; DC = dendritic cells; G = granulocytes; MF = macrophage; MC = monocyte; NK = natural killer cells; PC = precursor cells; SC = stem cells; T = T-cells; gdT = gamma-delta T-cells). The right margin lists p-values obtained by comparing expression in lung-derived DCs (DC.103-11b+.PolyIC.Lu) to expression in all other cell populations. Parts **B.** – **E.** show gene set enrichment analyses for GHRKO-decreased DEGs in BAT, EPI, PERI and SubQ, respectively. Genes are ranked from left to right according to how specifically they are expressed in lung-derived DCs (i.e., p-values from part A; left: most-specific; right: least-

specific). For each rank, the vertical axis tracks cumulative overlap between GHRKO-decreased DEGs and the ranked gene list. The level of enrichment is proportional to the area between the cumulative overlap curve and the diagonal, with positive area indicating that DEGs are enriched among genes specifically expressed in lung-derived DCs (e.g., part B). P-values in each panel are generated from the comparison between ranks of DEGs and all other depot-expressed genes (Wilcoxon rank sum test).
